# Display performance and standards for primary digital pathology sign-out: Technical specifications, validation, and quality assurance

**DOI:** 10.1016/j.jpi.2026.100670

**Published:** 2026-05-11

**Authors:** Yuqing Xiong, Sahussapont Joseph Sirintrapun

**Affiliations:** Mass General Brigham, Department of Pathology, Boston, MA, USA

**Keywords:** Digital pathology, Whole-slide imaging, Display monitors, Luminance, Pixel pitch, Refresh rate, Color fidelity, Quality assurance, Validation, Pixel pathway

## Abstract

**Context:**

As labs transition from glass slides to whole-slide imaging (WSI), the diagnostic display becomes the pathologist's new microscope and the terminus of the pixel pipeline.

**Objective:**

To synthesize technical, regulatory, and quality assurance (QA) guidance for monitors used in primary digital pathology sign-out and to clarify where pathology requirements diverge from radiology.

**Design:**

Narrative synthesis of available technical specifications from published technical articles for diagnostic displays in digital pathology, consolidated into practical guidance for selecting and interoperable swapping displays in the pixel pipeline.

**Results:**

Critically important monitor attributes include resolution/pixel density, luminance stability, contrast performance, and color rendering (gamut and bit depth). Debates remain over whether purpose-built medical-grade (MG) displays or high-performance consumer/professional-grade (CPG) displays are sufficient for digital pathology sign-out. Moreover, among the CPG display options, in-plane switching displays with light-emitting diode backlights remain preferred for sign-out over OLED (organic light-emitting diode) monitors. Whereas OLED monitors offer excellent contrast, there are durability and uniformity concerns. QA in labs deploying digital pathology should address luminescence deterioration and ambient lighting. Ergonomically, a 27-in. 4K display is adequate for primary sign-out; larger screens (30–32 in.) can expand the field of view but may introduce head/eye-movement and viewing distance burden.

**Conclusions:**

Pathology's reliance on polychromatic stains necessitates display standards distinct from radiology's grayscale focus. Validated CPG displays can be acceptable alternatives to universally mandating for digital pathology sign-out, costly MG displays, provided a disciplined QA program supports them. Rigorous local validation and ongoing QA are indispensable.

## Introduction

For over a century, the light microscope has been the definitive tool for anatomic pathologists. The advent of whole-slide image (WSI) catalyzed a paradigm shift from glass slides to digital images; in this new paradigm, the pathologist's workstation—and specifically the display—functions as the microscope.^1^ The integrity of every primary diagnosis rendered digitally is contingent on the monitor's ability to faithfully and consistently reproduce subtle morphological and tinctorial details that are interpretable for pathologists and pathology staff.

**Fig. 1:**
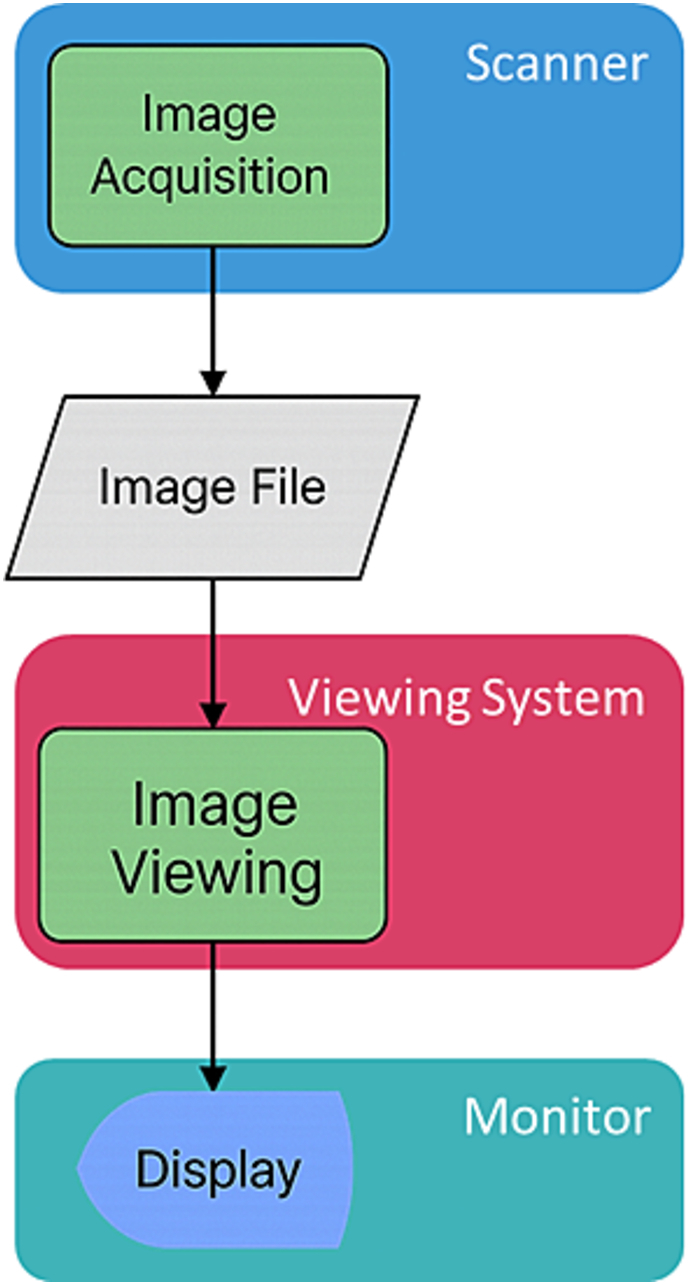
Digital pixel pipeline.

The U.S. Food and Drug Administration (FDA) does not approve or clear individual monitors for digital pathology as stand-alone devices. Instead, the FDA evaluates the end-to-end WSI system—the scanner, image file, viewing software, and specified display—as an integrated unit (the “pixel pipeline”) (see Fig. 1). Labs that substitute a non-specified display effectively modify the cleared system and must perform in-house validation and quality assurance (QA) under CLIA ‘88 as a laboratory-developed test (LDT).^2^ Importantly, this framework does not replace or diminish the role of the pathologist. Diagnostic interpretation remains the ultimate determinant of system adequacy, as the display must faithfully render features required for accurate clinical decision-making. During FDA clearance of WSI systems, pathologist performance is integral to validation studies, including assessment of display configurations within the end-to-end pixel pathway. In this context, the display specifications associated with FDA-cleared systems can be understood as reflecting thresholds of acceptable diagnostic performance established through pathologist interpretation. Accordingly, a specification-based approach to display selection does not “usurp” pathologist judgment, but rather operationalizes it into reproducible, scalable benchmarks that can be applied across diverse practice environments while preserving flexibility in implementation.

Radiology often prioritizes resolution, luminance, contrast offered in purpose-built medical-grade (MG) displays to ensure diagnostic accuracy for DICOM (Digital Imaging and Communications in Medicine) grayscale standard compliance.^3^ Digital pathology predominantly presents static brightfield images; beyond color fidelity, adequate luminance and resolution, very high resolution and contrast confer little diagnostic advantage over what is offered in high-performance consumer/professional-grade (CPG) displays. Likewise, pixel pitch gets handled differently from radiology. Whereas radiology uses a fixed pixel pitch (≈0.200 mm) linked to viewing distance for fixed-resolution images, pathology continuously adjusts effective resolution through zoom. Consequently, high pixel density (4 K or higher) in current CPG displays avoids pixelation across magnifications and is more impactful than adhering to a single nominal pixel pitch.

“Light-emitting diode (LED) monitors,” in common parlance, are liquid-crystal displays (LCDs) illuminated by LED backlights; they offer efficient, bright, and long-lasting illumination. IPS (in-plane switching) LCDs provide wide viewing angles and stable, accurate color, making them the current suitable “workhorse” display for digital pathology signout. By contrast to LED CPG displays, OLED monitors emit light per pixel and deliver superb contrast and “true black(s)” but carry risks of image retention (burn-in) and differential subpixel aging (notably blue), which can introduce long-term color shifts—undesirable in a diagnostic environment.^18^ “True black,” as achieved in OLED displays, refers to the absence of light emission in dark image regions, where individual pixels can be completely turned off, producing effectively infinite contrast. In contrast, LCD-based displays rely on a backlight and cannot achieve absolute black, resulting in slightly elevated black levels. However, medical imaging display standards do not require “true black”; instead, displays are evaluated based on measurable luminance characteristics such as minimum luminance (Lmin), maximum luminance (Lmax), and luminance ratio. This reinforces that diagnostic adequacy is defined by performance metrics rather than display technology, supporting a specification-based approach to validation.

This article will detail the known technical specifications sufficient for a primary diagnostic display in digital pathology, drawing parallels with and, more importantly, highlighting critical distinctions from the well-established standards in digital radiology. We will outline critical technical specifications for pathology displays and compare MG and high-performance CPG options. This article also highlights the specification gaps in displays for digital pathology. We will explore the regulatory landscape, analyze the display specifications of FDA-cleared systems, and provide a framework for validation and ongoing QA.

## Distinctions from radiology: Why pathology is a different challenge

Whereas digital pathology can learn from decades of experience in digital radiology, we believe that display requirements for radiology and pathology are NOT identical and that pathology should navigate these differences. Fundamental differences lie in the image characteristics rendered by radiology and pathology.

### Color vs grayscale

The most apparent distinction is pathology's reliance on color. The diagnosis of disease from hematoxylin and eosin (H&E), special stains, and immunohistochemistry (IHC) depends on the precise interpretation of a broad spectrum of colors and subtle gradients—the various shades of the deep purples of hematoxylin, pinks of eosin, and the rich browns of DAB chromogen are only some of the colors seen in pathology. Radiology, conversely, is primarily a grayscale discipline. Its standards, such as the DICOM Part 14 Grayscale Standard Display Function, are optimized for ensuring the perceptibility of subtle differences in luminance (shades of gray) and are not directly applicable to the polychromatic complexity of pathology.

### Pixel pitch and interaction model

In radiology, a strict pixel pitch (the distance between pixel centers, typically ∼0.200 mm) is often recommended, mainly because radiological images (e.g., a CT slice) have a fixed, finite resolution, and the standard establishes an optimal viewing distance for perceiving all available detail.^4^ WSIs in pathology, however, are different. WSIs are larger, comprising of enormous datasets, often exceeding 10 gigapixels, that pathologists interact with through continuous panning and zooming, with constant changing of the image resolution on the screen. Therefore, a single, fixed pixel pitch is less relevant than having a very high overall resolution (i.e., a high pixel density) that allows for crisp, precise details across a wide range of magnifications without pixelation.

### Contrast and black levels

Radiologists require exceptional contrast and actual black levels to discern faint nodules or subtle density changes in grayscale images. Whereas comparison is also essential in pathology for tasks like identifying hyperchromatic nuclei, there is less need in pathology for the “perfect black”. Because the diagnostic information for pathology is conveyed through color vs grayscale, the “imperfect” blacks of modern LCD technology in CPG displays do not typically impede the interpretation of a brightfield H&E image rendered in WSIs.

## Critical technical specifications and considerations for pathology displays

### Resolution, pixel density, screen size, and refresh rate

High resolution allows simultaneous assessment of tissue architecture and cytological detail. Prior studies and industry have recommended a minimum of 4 MP is a baseline; 8 MP (3840 × 2160, 4 K) as optimal for maximizing image detail and workflow efficiency.^5^, ^6^, ^7^ A 27-in. 4 K monitor is adequate and ergonomically favorable for digital pathology sign-out. Some teams prefer 30–32 in. to widen the field of view and decrease panning; however, beyond ∼32 in., increased head/eye movement and increased viewing distance may offset gains.^8^ An 8 MP display may allow clear visualization of nuclear membrane irregularities, chromatin patterns, and nucleoli without zooming in so closely that the surrounding architectural context is lost; thus improving both diagnostic accuracy and workflow efficiency. Finally, because digital pathology primarily deals with static images, the refresh rate has minimal diagnostic impact once the WSI is loaded. Pathologists interact with images through panning and zooming, which benefit more from robust graphics processing than from an ultra-high refresh rate. Thus, a standard refresh rate of 60 Hz would be sufficient.

### Luminance, contrast, monitor technology, and ambient lighting

A minimum calibrated luminance of 300 cd/m^2^, with 400–500 cd/m^2^ preferred for brightfield fidelity and headroom as the backlight ages.^9^, ^10^ Ideally, this luminance remains stable over the display's lifespan. Static contrast ≥1000:1 is recommended and considered the standard benchmark for IPS LCD monitors for balancing image quality, deep blacks, and bright whites for daily tasks; emerging IPS Black variants (∼2000:1) can further enhance shadow detail by rendering deeper blacks. IPS LCD remains the standard due to superior color accuracy and consistency, and viewing angles (≈178°), supporting collaboration and tumor boards.^10^

In radiology and the grayscale nature of radiological images, ambient lighting is critical. Whether ambient lighting is also critical in pathology still requires some additional study with diagnostic performance and interplay between luminance and ideal ambient lighting levels. For radiology, optimum ambient lighting seems to be around 25–40 lx at luminance ≥ ∼350 cd/m^2^ (or ∼420 cd/m^2^ for mammography).^11^, ^12^ Some sources for mammography-specific reading rooms have suggested even lower levels, e.g., ambient <10 lx for displays of 500–600 cd/m^2^.^13^ What is known is that there are no recommendations for how to manage or mitigate ambient light for digital displays in pathology and that easy user adjustment of luminance is preferable for adapting to various ambient light conditions. Moreover, to minimize glare and variable illumination, the recommendation is to position displays perpendicular to windows with the use of blinds and curtains to control natural light. The positioning of displays directly opposite or adjacent to windows without blinds or curtains should be avoided.^14^

### Color fidelity: Gamut and bit depth

Color is the most distinguishing parameter separating pathology from radiology. The monitor must faithfully reproduce the colors of the lab's specific stains. Because diagnostic information is encoded in color, the display should cover ≥100% of the sRGB color space with an accurate 10-bit pipeline. An 8-bit display can show 16.7 million colors, but a 10-bit display can show 1.07 billion colors. This vastly expanded palette is essential for rendering the smooth, subtle gradients seen in IHC staining, preventing “color banding” artifacts that can obscure the difference between weak-positive and negative results. Whereas the number of colors discernible by the human eye is often cited in discussions of display requirements, the rationale for a 10-bit color pipeline in digital pathology is not based solely on exceeding human perceptual limits. Rather, higher bit depth reduces quantization artifacts and preserves smooth gradients across the entire pixel pathway. WSIs undergo multiple processing steps—including scanner acquisition, compression, color transformation, rendering, and GPU output—each of which may introduce rounding error when constrained to lower bit depths. Supporting a 10-bit pipeline minimizes cumulative precision loss and ensures that subtle chromatic differences are faithfully preserved at the point of display. This is particularly important in histopathology, where diagnostically relevant features may depend on faint, low-intensity staining patterns. In this context, 10-bit color should be viewed not as exceeding human vision, but as preserving signal fidelity across a complex digital workflow. For example, one study looked at the concordance of HER2 between microscopy and digital across multiple types of monitors. Whereas the concordance was 100% for HER2, the authors noted the limitations because they excluded HER2-low cases.^15^ These are the cases, where it is important to have sensitive displays to characterize the nuances of the faint and incomplete membranous staining of HER2. New clinical trials have shown that HER2-low and HER2-ultralow patients can still benefit from therapy, highlighting the importance of accurate quantification and diagnosis.^16^ In HER2-low assessment, faint incomplete membrane staining must be distinguished from absence of staining, staining in too few cells, or non-specific/background signal. That makes faithful rendering of subtle brown chromogen intensity and membrane localization important, especially near the 0-vs-1+ threshold where treatment implications now exist. In this setting, display-related artifacts such as color banding, limited bit depth, or inaccurate color rendering may obscure or exaggerate low-intensity chromogen signal, potentially affecting the distinction between true faint membranous staining and background. This is particularly relevant in the HER2-low context, where small perceptual differences may alter categorization and, consequently, patient eligibility for targeted therapies. Thus, display performance becomes not only a technical consideration but also a clinically consequential component of the digital pathology pipeline.

Lab-specific stain appearance should be evaluated during validation. Determining the “gold-standard” specifications for color in digital pathology is a moving target. Pathology labs over the past century continue to produce slides that are stained variably in purple and pink with H&E, along with other color variations with special stains and IHC. For pathologists working at these lab sites, no legal cases demonstrate that such routine staining variability has led to diagnostic misinterpretation detrimental to patient care. Pathologists “adjust” to local lab color variability and can also adjust to other labs when receiving outside slides for diagnostic interpretation. Furthermore, color-blind pathologists have practiced for as long as labs have produced stained glass slides. Mandating a single uniform color standard for every lab would provoke pathologists to revolt against what they are accustomed to routinely in their practice and would constitute regulatory overreach unlikely to be sustained. That is not to say that color standardization is unachievable. Measures like mandating that digital scanner vendors scan in accordance with International Color Consortium color profiles are a reasonable, bare minimum step. Though extra steps, such as leveraging color QA tools like a Sierra slide and analyzers are available, their sustained implementation complexity make them feasibly impractical at every under-resourced pathology lab.^17^ Moreover, it remains unclear whether such QA measures are necessary to avoid diagnostic misinterpretation detrimental to patient care.

## Monitor technology: LCD vs OLED

### Liquid-crystal displays (LCDs)

LCDs are a flat monitor display technology that uses electrically charged liquid crystals to create images. These displays are standard in devices like televisions, computer monitors, and smartphones and function by using liquid crystals to block or allow light from a backlight to pass through, forming images. Early LCDs used cold cathode fluorescent lamps. In contrast, modern “LED” screens use LEDs for backlighting, offering advantages such as better picture quality, thinner designs, improved energy efficiency, greater stability, longer lifespan, and color accuracy. Minor backlight bleed and imperfect blacks rarely impact a color-rich, brightfield interpretation.^18^

### Organic light-emitting diode (OLED)

Whereas displays with OLED technology offer superior contrast ratios and perfect blacks over LCDs, OLED displays are not yet ideal for the pathology use case. As an emissive technology, each pixel generates its own light. OLED allows for unparalleled contrast but comes with significant drawbacks for a pathology workstation, including: (1) permanent burn-in from static UI elements/toolbars; (2) uneven degradation and color drift over time, most notably blue subpixels; and (3) potential non-uniformity at low luminance.^19^ These durability concerns currently limit OLED suitability for routine primary diagnosis pending mitigations. Until these issues are resolved, high-quality IPS LCDs remain the most reliable and appropriate technology for primary diagnosis in pathology (see Table 1).

**Table 1:** LED, LCD, and OLED—features and pathology relevance.

Technology	Core concept	Pros for pathology	Cons / Risks
LED-backlit LCD (IPS)	Transmissive liquid crystal with LED backlight	Stable color, wide viewing angles, long life, minimal image persistence	Imperfect blacks; potential backlight bleed; requires periodic calibration
Mini-LED LCD	Dense local-dimming zones behind LCD	Higher peak luminance, improved contrast uniformity	Haloing possible; complexity; cost
OLED	Emissive per-pixel light	Superb contrast/black; fast response	Burn-in risk; blue subpixel aging → color drift; uniformity over time

## Medical-grade - vs consumer (professional-grade) displays

The choice between a purpose-built MG display and a high-performance CPG display is a strategic decision about risk and resource management (see Table 2).

**Table 2:** Medical-grade (MG) vs consumer/professional-grade (CPG) displays for digital pathology sign-out.

Attribute	Medical-grade (MG) display	Professional-grade (CPG) display
Typical monitor	IPS LCD (with medical firmware)	IPS LCD (creator/graphics class)
Backlight & stabilization	LED backlight with built-in luminance stabilization; front sensor for auto-QA	LED backlight; no built-in stabilization; requires external meter/software
Factory calibration	DICOM-GSDF and/or vendor medical preset; documentation provided	Creator presets (sRGB/Rec.709/DCI-P3); documentation varies
QA workflow	Automated (scheduled, unattended) with audit logs	Manual (scheduled by lab) with external colorimeter and logs
Uniformity compensation	Hardware-level digital uniformity equalizer	Varies by model; often present but not validated for medical use
Warranty/lifecycle	5+ years typical; vendor support SLAs	3 years typical; standard commercial support
Certifications	IEC/EN medical safety; often FDA-listed as part of the WSI system	Commercial certifications only
Burn-in mitigation	Not applicable to LCD; vendors monitor drift proactively	Not applicable to LCD; drift handled by lab QA
Cost (relative)	High	Moderate to low
Suitability	Turn-key in regulated settings with limited physics/IT support	Viable when the lab can own robust QA and validation

*Note:* OLED monitors are generally avoided for primary sign-out due to image retention and color-shift risks over time.

MG monitors are purpose-built for clinical imaging (e.g., integrated front sensors, auto QA, luminance stabilization, extended warranties, and medical certifications). Their premium cost is justified by integrated features such as front sensors that provide automated, continuous QA, ensuring luminance and color remain stable and calibrated over time. Such QA tools avert the manual routine use of photometers, which require significant resourcing by departments and their institutions. They also carry medical-specific certifications and are built for a longer operational lifespan with extensive warranties (≥5 years). In radiology, support teams, like the scalability in deployment and QA across sites, include remotely at radiologists' homes.

CPG monitors, high-end graphics/creator monitors (e.g., IPS 4 K) often used in graphical design fields or competitive gaming, may meet or exceed required specifications for pathology at a lower cost but lack integrated QA,“ or “CPG monitors are high-end graphics/creator panels (e.g., IPS 4 K) often used in graphical design fields or competitive gaming and may meet or exceed required specifications for pathology at a lower cost but lack integrated QA. When used in cleared systems or as LDTs, the lab must implement disciplined manual QA with possible external colorimeters and software. The FDA clearance of the Sectra Digital Pathology Module with the Dell U3223QE has validated this as a viable option. Still, it requires the lab to commit to a rigorous, ongoing manual QA program (see Table 3). Another option is to replace the lab's monitors every few years. Interestingly, studies have shown that there is not much diagnostic difference between MG displays and CPG displays.^5^, ^6^, ^7^

**Table 3:** Monitor quality assurance (QA) program—suggested schedule and checks.

Frequency	Check	Tool/Method	Acceptance/Action
At installation	Baseline luminance & color calibration; uniformity map; visual test pattern review	External colorimeter + vendor/third-party QA software;	At baseline from manufacturer; replace if not
Daily (startup)	Quick visual check of test pattern; UI/readability	Built-in pattern or printed reference image	If artifacts, restart app/GPU; log issue
Monthly	Luminance spot check at center; color drift check	External colorimeter (short run)	Recalibrate if luminance drift shows difference compared to glass
Quarterly	Full-field uniformity (center +4–9 points); gamut verification	QA software uniformity routine	Service if non-uniformity
Annually	Full calibration + documentation; review ambient lighting	External colorimeter; lux meter	Update and compare all specifications to baseline; consider replacing if needed
Lifecycle (3–5 yrs)	Replacement planning	Procurement + physics/IT	Replace when unable to meet thresholds despite calibration

*Notes:* Perform QA under typical reading conditions (lights, blinds, wall color). Keep audit logs for CLIA/CAP documentation.

## Validation and quality assurance

Regardless of the monitor chosen, the lab bears the ultimate responsibility for proving it is fit for purpose within its specific environment. In practice, validation is performed at the level of the display model or, more broadly, against defined technical specifications, rather than on each individual monitor deployed within an institution. It is neither practical nor necessary to validate every unit in a fleet, provided that monitors are procured from the same model line and perform within manufacturer-defined tolerances. More importantly, a specification-based validation approach—aligned with display characteristics derived from FDA-cleared WSI systems—decouples validation from specific hardware models. This enables scalable enterprise deployment and future-proofs procurement, as individual display models inevitably evolve or are discontinued. Under this framework, replacement displays may be introduced without full system re-validation, provided they meet the validated performance benchmarks across the digital pathology pixel pathway.

As per the College of American Pathologists (CAP) guidelines, the lab must conduct an intraobserver concordance study.^1^ Such a concordance study involves reviewing a set of at least 60 cases, representative of the lab's workload, first on a light microscope and then on the digital system after a “washout period” of at least 2 weeks. A diagnostic concordance of >95% must be achieved and documented. The Laboratory Medical Director must sign off on this entire process before the system can be used for patient care (see Table 4).

**Table 4:** Example acceptance template for intraobserver validation (≥60 cases).

Element	Requirement	Recorded value
Case mix	Representative across subspecialties (biopsies, resections, cytology, if applicable)	
Washout period	≥2 weeks between glass and digital reads	
Concordance target	≥95% primary diagnostic concordance	
Major discordances	0 tolerated without documented root cause/mitigation	
Reader training	Documented training on the viewer and display	
Environment	Ambient lighting, neutral walls, no glare	
Final approval	Medical Director signs-off before clinical use	

Most importantly, a monitor's performance degrades over time. An ongoing QA is essential to ensure the display remains in its validated state. For MG displays, this can be automated with built-in features such as advanced technologies for color calibration, brightness stabilization, and uniformity correction. For CPG displays, this requires a manual protocol for periodic checks and recalibration using external hardware, as mentioned earlier. This process must be performed in the pathologist's actual reading environment, as ambient lighting can significantly affect image perception.

External calibration tools, such as colorimeters and photometers, are used to quantify luminance, color accuracy, and uniformity during validation and periodic recalibration of displays. These tools generate objective measurements—such as luminance output, contrast ratio, and color deviation—that enable labs to monitor drift and maintain consistency over time. However, the clinical significance of these measurable differences remains uncertain. Studies have shown minimal differences in diagnostic accuracy between calibrated MG and high-performance consumer-grade displays, even when technical differences are present. These differences should also be interpreted in the context of intrinsic variability in histological staining, which can vary significantly across labs and over time. Pathologists routinely accommodate such variability in routine practice, suggesting that modest deviations in display calibration are unlikely to meaningfully impact diagnostic interpretation. In this context, calibration serves primarily to ensure consistency and reproducibility within a given digital pathology workflow, rather than to enforce a rigid or universal display standard.

Although relatively few studies have directly evaluated diagnostic accuracy across different display types (i.e., MG vs CPG), prior work suggests that MG displays may demonstrate improved concordance with microscopy for H&E slides when assessing parameters such as image clarity, color fidelity, and luminance stability.^15^ This likely reflects the impact of integrated calibration tools designed to maintain consistent display performance over time. However, despite measurable technical differences, clear differences in diagnostic accuracy between calibrated MG displays and well-configured CPG displays have not been consistently demonstrated.^15^ In contrast, some studies have reported differences in interpretation efficiency, with statistically significant variations in reading speed across display types.^20^ Increased interpretation time may contribute to user fatigue, which could indirectly influence diagnostic performance in high-volume workflows. Importantly, these differences should also be interpreted in the context of inherent variability in histological staining, which can vary across labs and over time. Pathologists routinely adapt to such variability in clinical practice, suggesting that modest differences in display calibration may not translate into meaningful diagnostic impact. Accordingly, calibration and QA efforts should focus on maintaining consistency and stability within a given system rather than achieving a singular, idealized display standard.

## Discussion

The display monitor is a mission-critical component of any digital pathology system. Whereas drawing lessons from radiology, labs must recognize that pathology's reliance on color introduces unique and demanding requirements. The selection of a display should be guided by the performance benchmarks set by monitors included in FDA-cleared WSI systems. Many issues are still under discussion, such as luminescence deterioration, monitor size, color fidelity, ambient lighting, and the level of QA that is feasibly deployed across institutions.

Declining luminance can compress perceived dynamic range and obscure weak staining. Mitigations could include minimum brightness thresholds, drift tracking, auto- or manual calibration, and proactive replacement of monitors. Regular luminance checks are vital, as dimmer displays can mask subtle variations in stain.

IEC 62563 is the International Electrotechnical Commission (IEC) standard for *Image Quality Evaluation* of medical imaging display systems. It was initially developed for radiology monitors but is now increasingly referenced for digital pathology because of its structured framework for testing, acceptance, and ongoing QA. IEC 62563 is increasingly used as the de facto framework for QA of displays in digital pathology, given the absence of a pathology-specific international standard. The standard defines structured procedures for acceptance testing and ongoing constancy checks, including evaluation of luminance response, maximum luminance, uniformity, resolution, noise, pixel defects, and the impact of ambient light. Although its luminance and grayscale requirements were designed for radiology, the underlying methodology is readily adapted to digital pathology, where higher luminance levels, color accuracy, and controlled—but not dark-room—ambient lighting conditions are required. Display manufacturers and medical institutions commonly apply IEC 62563 as a QA benchmark for digital pathology workstations, ensuring consistent visual performance, supporting regulatory compliance, and providing a standardized approach to monitor drift and long-term display quality, which are essential for safe diagnostic use. As rigorous as these approaches are, the critical question remains whether all under-resourced labs can implement them to assure QA at these levels. Note that such approaches require skilled personnel with photometers; resources that nearly all labs lack. In addition, the question remains whether such a level of rigor is necessary for diagnosing with digital pathology. Regardless, a highly anticipated update may provide recommendations that labs can feasibly deploy across all pathology labs.^21^

### Recommendations

Much of monitor size selection may depend on the user and workspace; we suggest a minimum 27-in. 4 K monitor is broadly adequate and ergonomically balanced for primary sign-out based on our analysis and prior studies. Larger 30–32-in. displays can increase context at a glance and reduce panning, but may increase head/eye movement, cost more, and, because of increased viewing distance, be impractical due to space limitations. Selection should consider workstation layout and user preference.

Whereas color accuracy matters, insisting on uniformly expensive, tightly calibrated MG displays for all use cases may overcompensate for clinical risk. Pathologists primarily assess relative color relationships and morphology. We believe CPG displays, when implemented with proper QA protocols and reasonable calibration, can achieve reliable performance without an unnecessary financial burden. Investments, therefore, can prioritize validation rigor and QA processes over maximal hardware specifications alone.

In enterprise digital pathology deployments, standardization of display specifications is generally preferred to ensure consistency with the configuration used during validation. A standardized baseline supports reproducible diagnostic conditions, simplifies QA, and facilitates scalable deployment. However, a specification-based framework—defined by performance parameters such as luminance, contrast ratio, resolution, and color calibration—can allow limited flexibility for alternative display configurations that meet the same validated benchmarks. Under this approach, non-standard displays (e.g., larger or curved monitors) may be accommodated through calibration or targeted non-inferiority assessments rather than full system re-validation, provided equivalent performance is demonstrated across the digital pathology pixel pathway. Practical considerations remain important: larger displays may introduce ergonomic challenges related to viewing distance and eye movement, whereas curved displays may introduce perceptual or luminance non-uniformity across the screen. Accordingly, while individual preference can be considered, maintaining standardized, validated display specifications across the enterprise provides the most reliable and scalable model. Thus, in conclusion, for primary digital pathology sign-out, we recommend an IPS LCD with 4 K resolution (≥8 MP), a minimum calibrated luminance of ≥300 cd/m^2^ (preferably 400–500 cd/m^2^), contrast ≥1000:1, ≥100% sRGB coverage, and an accurate 10-bit pipeline (see Table 5). Many of these specifications are derived from FDA approved MG monitors for digital pathology, such as the Barco MDPC-8127.^22^ A 27-in. form factor is sufficient for routine sign-out; 30–32 in. may be beneficial where desk space and ergonomics allow. The decision between MG and professional-grade monitors should depend on the lab's QA infrastructure and financial constraints. Ultimately, rigorous validation and maintenance, rather than over-engineered hardware, ensure the safety, accuracy, and reproducibility of digital pathology workflows.

**Table 5:** Recommended display specifications for primary digital pathology sign-out.

Parameter	Recommendation	Rationale
Resolution (pixels)	≥3840 × 2160 (4 K, ∼8 MP)	Enables wide field of view and fine cytology without excessive zooming
Screen size	27 in. (adequate); 30–32 in. (optional for larger FOV)	Balances context vs head/eye movement; ergonomics
Pixel density	≥160 ppi (typical for 27-in. 4 K)	Minimizes pixelation across zoom levels
Luminance (calibrated)	≥300 cd/m^2^ (preferred 400–500 cd/m^2^)	Brightfield fidelity and headroom for aging/backlight drift
Contrast ratio (static)	≥1000:1 (IPS Black ∼2000:1 beneficial)	Improves separation of lightly stained structures
Color gamut	≥100% sRGB coverage	Matches stain appearance workflows
Color depth	True 10-bit pipeline end-to-end	Reduces banding; preserves subtle IHC gradients
Viewing angles	178° horizontal/vertical (IPS)	Multi-viewer consistency; tumor boards
Uniformity	Luminance/color uniformity compensation preferred	Reduces edge/center variance at low magnification
Refresh rate	≥60 Hz	Adequate for static WSI; higher rates add little diagnostically
Connectivity	DisplayPort/USB-C (10-bit capable), GPU support	Ensures 10-bit output and stable timing
Ambient light	Sufficient lighting, monitor perpendicular to window, curtains/blinds near windows	Controls glare and perceived contrast

## Declaration of competing interest

The authors declare that they have no known competing financial interests or personal relationships that could have appeared to influence the work reported in this paper.
